# Extracellular HSP70/HSP70-PCs Promote Epithelial-Mesenchymal Transition of Hepatocarcinoma Cells

**DOI:** 10.1371/journal.pone.0084759

**Published:** 2013-12-27

**Authors:** Hangyu Li, Yan Li, Dan Liu, Hongzhi Sun, Dongming Su, Fuquan Yang, Jingang Liu

**Affiliations:** 1 Department of General Surgery, Shengjing Hospital Affiliated to China Medical University, Shenyang, China; 2 Department of General Surgery, First Hospital Affiliated to Liaoning Medical College, Jinzhou, China; 3 Center of Metabolic Disease Research, Nanjing Medical University, Nanjing, China; Cincinnati Children's Hospital Medical Center, United States of America

## Abstract

**Background:**

Extracellular heat shock protein 70 and peptide complexes (eHSP70/HSP70-PCs) regulate a variety of biological behaviors in tumor cells. Whether eHSP70/HSP70-PCs are involved in the epithelial-mesenchymal transition (EMT) of tumor cells remains unclear.

**Aims:**

To determine the effects of eHSP70/HSP70-PCs on EMT of hepatocarcinoma cells.

**Methods:**

The expressions of E-cadherin, HSP70, α-smooth muscle actin protein (α-SMA) and p-p38 were detected immunohistochemically in liver cancer samples. Immunofluorescence, western blotting and real-time RT-PCR methods were used to analyze the effects of eHSP70/HSP70-PCs on the expressions of E-cadherin, α-SMA and p38/MAPK in vivo.

**Results:**

HSP70, E-cadherin, α-SMA and p-p38 were elevated in hepatocellular carcinoma tissues. The expression of HSP70 was positively correlated with malignant differentiated liver carcinoma. The expressions of HSP70, α-SMA and p-p38 correlated with recurrence-free survival after resection. eHSP70/HSP70-PCs significantly promoted the expressions of α-SMA and p-p38 and reduced the expressions of E-cadherin in vivo. The effect was inhibited by SB203580.

**Conclusion:**

The expressions of HSP70, E-cadherin, α-SMA and p-p38 may represent indicators of malignant potential and could discriminate the malignant degree of liver cancer. eHSP70/HSP70-PCs play an important role in the EMT of hepatocellular carcinoma via the p38/MAPK pathway.

## Introduction

Primary liver cancer is the world's sixth highest incidence malignancy and its mortality is the third highest worldwide [1]. Approximately 55% of the incidence of liver cancer occurs in China [2]. Currently, surgery is the best treatment for primary liver cancer, but tumor metastasis and relapse seriously hamper the long-term survival of patients. Therefore, it is necessary to characterize the molecular mechanism of liver cancer metastasis. 

Invasion by tumor cells is the premise of tumor metastasis, and epithelial-mesenchymal transition (EMT) plays an important role in tumor cell invasion and metastasis [3]. EMT is a complex process that refers to the transformation of epithelial cells to stromal cells, in which the polarity of epithelial cell disappears, accompanied by enhanced migration and invasion[4]. An important characteristic of EMT is the loss of expression of epithelial cell markers (cadherin and E-cadherin) and/or overexpression of mesenchymal cell markers (e.g., α-smooth muscle actin protein, α-SMA) [4]. EMT occurs during the development and progression of liver cancer and is closely related to the invasion and metastasis of liver cancer [5,6]. The occurrence of liver cancer is closely related to its microenvironment [7]. A variety of factors in the tumor microenvironment could induce EMT, including hepatocyte growth factor (HGF), fibroblast growth factor (FGF), and platelet-derived growth factor (PDGF) [8]. Heat shock protein 70 and HSP70 peptide complexes (HSP70/HSP70-PCs) are also present in the tumor microenvironment [9]. The HSP70 protein family is involved in the regulation of many biological processes of tumors [10]. In addition, extracellular HSP70/HSP70-PCs (eHSP70/HSP70-PCs) can act directly on tumor cells to regulate a variety of their functions, including proliferation [11] and invasion [12]. Whether eHSP70/HSP70-PCs are involved in the EMT of tumor cells remains unclear. 

EMT results from a variety of extracellular signaling molecules through cascade activations of downstream proteins. The mitogen-activated protein kinase (MAPK) signaling pathway is the common extracellular signal pathway that causes the nuclear reaction and the p38/MAPK signal pathway is involved in the occurrence of EMT [13]. p38 is involved in the regulation of a variety of biological processes in tumor cells, including proliferation, invasion and apoptosis [13]. Under exposure to ionizing radiation, lung cancer cells showed EMT-related morphological changes, along with the activation of p38/MAPK, with no activation of extra cellular regulated protein kinases 1/2 (ERK1/2) or c-Jun N-terminal kinase (JNK) [14]. Our study aimed to investigate whether extracellular HSP70/HSP70-PCs induced EMT in hepatoma cells, and whether p38/MAPK was involved this induction. 

In this study, we studied the expressions of E-cadherin, α-SMA and p-p38 proteins in liver cancer tissues and their relationship with clinicopathological parameters. We further investigated the mechanism of the effect of eHSP70/HSP70-PCs on EMT in Huh-7 hepatoma liver cells. 

## Materials and Methods

### Ethical statement

The Institutional Review Board of the Shengjing Hospital of China Medical University approved the use of human tissue samples for this project. All patients gave their informed and written consent for the use of the clinical specimens for research. 

### Patient samples and patients’ demographic data

The study included 102 patients undergoing surgical removal of liver cancer from March 2008 to September 2010 in Shengjing Hospital of China Medical University. Sixty pairs of HCC samples and their corresponding non-tumorous liver tissues were employed for real time-quantitative PCR (qPCR) and western blot analysis. The clinicopathological features of the patients are summarized in Table 1. The resected specimens were obtained immediately after surgical resection, snap-frozen in liquid nitrogen, and kept at -80°C. The non-tumorous liver samples were excised from tissue more than 1 cm from the tumors.

**Table 1:** The relationship between expression of α-SMA, HSP70, E-cadherin and p-p38 and clinicopathological parameters in hepatomas.

	n	α-SMA			E-cadherin			p-p38			HSP70			
		low	high	***p***	low	high	***p***	low	high	***p***	low	moderate	high	***p***
Age														
>45	46	30	16	>0.05	27	19	>0.05	24	22	>0.05	16	17	13	>0.05
<45	56	39	17		30	26		36	20		24	22	10	
Sex														
Male	59	40	19	>0.05	37	22	>0.05	31	28	>0.05	20	25	14	>0.05
Female	43	29	14		20	23		29	14		20	14	9	
The stage of TNM														
I+II	65	50	15	>0.05	30	35	<0.05	48	17	<0.05	39	23	3	<0.05
III+IV	37	19	18		10	27		12	25		1	16	20	
Tumor														
>5cm	36	22	14	>0.05	19	17	>0.05	22	14	>0.05	16	14	6	>0.05
<5cm	66	47	19		38	28		38	28		24	25	17	
Degree of differentiation														
Well	43	37	6	<0.05	7	36	<0.05	34	9	<0.05	32	4	7	<0.05
Moderate	41	25	16		8	33		18	23		7	23	11	
Poor	18	7	11		1	17		8	10		1	12	5	
Lymph node metastasis														
Yes	35	49	18	>0.05	24	11	>0.05	10	25	<0.05	7	13	15	<0.05
No	67	20	15		33	34		50	17		33	26	8	
The portal vein invasion														
Yes	42	22	20	<0.05	35	7	<0.05	18	24	<0.05	7	24	11	<0.05
No	60	47	13		22	38		42	18		33	15	12	

### Immunohistochemical Staining

All specimens were routinely fixed in neutral-buffered formalin and embedded in paraffin. Serial 4-μm sections were cut from archived paraffin blocks for immunohistochemical study. Standard hematoxylin-eosin staining was used for routine histological examination. Briefly, the 4-μm-thick sections were deparaffinized and treated with 0.3% endogenous peroxidase blocking solution for 20 minutes. Sections were treated sequentially with normal goat serum for 20 minutes and incubated with anti-α-SMA antibody at a dilution of 1:200 for 1.5 hours at room temperature and then incubated with HRP-labeled goat-anti-rabbit secondary antibodies (diluted to 1:200). Samples were analyzed by confocal microscopy (200× magnification; Olympus, Tokyo, Japan). The expression and distribution of HSP70, E-cadherin and p-p38 in liver tissues were determined in the same way. The positive index (PI) was calculated as the mean optical density multiplied by the positive area percentage.

Yellow or brown particles were regarded as positive, using an integrated evaluation method for scoring criteria; in each slice, 100 cells were randomly observed. The dyeing range rating criteria were as follows: 0 score, no positive cells, 1 score, 0-30% positive cells, 2 scores, 31-60%, 3 scores, > 60%. Staining intensity scoring criteria were as follows: 0 score, no staining, 1 score, thin yellow particles for a pile, 2 scores, thick dark-yellow particles diffuse, 3 scores, thick brown particles diffuse. A score of 0-3 represented low-expression and a score of 4-6 represented high-expression [15].

We analyzed the relationship between the expression of E-cadherin, HSP70, α-SMA and p-p38 in HCC and the clinicopathological characteristics including TNM staging, lymph node metastasis, portal vein invasion, liver cancer differentiation degree and prognosis of patients.

### Extraction and purification of extracellular HSP70/HSP70-PCs

Hepatocarcinoma tissue samples were homogenized in lysis buffer (NaHCO_3_ 30 mmol/L pH 7.1, 0.1 mmol/L EDTA, 0.1 mmol/L DTT and 0.5 mmol/L PMSF) and centrifuged at 150000×g for three hours at 4°C, and the supernatant was collected. The supernatant was concentrated by PEG (MW 600) and applied to a ConA-sepharose column in the presence of ConA-sepharose binding buffer C (20 mM Tris-acetate, pH 7.5, 0.5 mM NaCl, 2 mM CaCl_2_, 2 mM MgCl_2_, 15 mM 2 ME and 0.5 mM PMSF), and the ConA-sepharose unbound fraction was collected at a flow rate of 12 ml /h. The fraction was dialyzed against buffer D (20 mM Tris-acetate, pH7.5, 20 mM NaCl, 3 mM MgCl_2_, 15 mM 2ME and 0.5 mM PMSF) overnight at 4°C. The harvested elute was concentrated and dialyzed against DEAE ion exchange buffer A (20 mM Na_3_PO_4_, 20 mM NaCl, pH7.2). The sample was applied to a DEAE column and equilibrated with buffer A at a flow rate of 10 ml/h. After buffer A equilibration for 30 min, the target protein was eluted using a linear gradient of 20 mM-1 000 mM NaCl in buffer A (20 mM Na_3_PO_4_, 1M NaCl, pH7.0, ranging from 0%-100%). The harvested fractions were subjected to SDS-PAGE and western blotting using a monoclonal antibody specific for HSP70 [16]. The fractions containing the HSP70 protein were collected, pooled and dried by freeze-drying, before being stored at -20°C until further use. 

### Cell culture

Human HCC cell line Huh-7 was purchased from Shanghai Institute of Cell Bank. The cells were cultured at 37°C in DMEM medium supplemented with 10% FBS for 2 to 3 days, with 0.25% trypsin digestion and passage. Logarithmic growth phase cells were used for the experiments. The cells were divided into the groups: the control group, the extracellular HSP70/HSP70-PCs (final concentration 2μg/ml) treated group, and SB-203580 (an p38 MAPK inhibitor, Sigma-Aldrich Co., St. Louis, MO, USA) with the extracellular HSP70/HSP70-PCs treated group. 

### Immunofluorescence Analysis

Huh-7 cells were treated with extracellular HSP70/HSP70-PCs , then fixed with 4% paraformaldehyde for 30 min and blocked in 1×PBS (pH 7.4) solution with 3% BSA. The anti-α-SMA (Abcam, Cambridge, UK) or anti-E-cadherin-antibodies (Abcam) were added at a dilution of 1:1000 and incubated overnight at 4°C in a humidified box. After washing, the fluorescent secondary antibody (Santa Cruz Biotechnology, inc, CA, USA) was added at a dilution of 1:100 and incubated for 2 h. The cells were then washed three times with1×PBS, and counter-stained with DAPI (Santa Cruz Biotechnology). Confocal microscopy was performed and fluorescence was analyzed using a fluorescence microscope (Leica DMI4000B, Buffalo Grove, IL, USA).

### Real-time RT-PCR

 Total RNA was isolated with the Trizol reagent (Invitrogen, Carlsbad, CA, USA) from the tissues and cells at 24 and 48 hour after induction, according to the manufacturer’s instructions. Single-strand cDNA was synthesized from 1μg of total RNA by reverse transcription according to the manufacturer’s instructions (Toyobo, Japan). Real-time PCR was used to measure the mRNA levels of E-cadherin, α-SMA, p38 in the tissues and cells. Quantitative PCR was performed on an ABI-7500 Sequence Detector (Applied Biosystems, Foster City, CA). Amplification was carried out in a 25-μl volume for 35 cycles and the product was detected using SYBR Green fluorochrome. The primers were as follows:

E-cadherin:
Forward: 5'-CCGCCATCGCTTACA -3'
Reverse: 5'-GGCACCTGACCCTTGTA-3'
α-SMA
Forward: 5'-CGGGACATCAAGGAGAAACT-3'

Reverse: 5'CCATCAGGCAACTCGTAACTCT3'

P38
Forward: 5'-GCTGAAGATTCTGGATTTTG-3'
Reverse: 5'-GTTCTTCCAGTCAACAGCTC-3'
GAPDH
Forward: 5'-GGATTTGGTCGTATTGGG-3'

Reverse: 5'-TCGCTCCTGGAAGATGG-3

### Western blotting

Western blotting was performed to confirm the results of immunohistochemistry. Proteins were extracted from tissues according to the instructions provided with the kits (Yafa, Wuhan, China). Forty-eight hours after induction, cells were harvested and prepared for immediate protein extraction. The extracted proteins were separated by 10% SDS-PAGE and then transferred to polyvinylidene fluoride membranes. After being incubated with 10% nonfat milk, the membranes were probed with α-SMA (Abcam) antibody (1:1000) overnight at 4 °C and then incubated with HRP-labeled secondary antibodies (Santa Cruz Biotechnology). Relative expression was quantified according to reference blots of GAPDH (Sigma).

### Statistical analyses

 The correlation between tumor protein expression and the clinicopathological features was performed using chi-square or Fisher exact tests. A paired-sample *t* test was used to compare the protein and mRNA expression in liver tumors with that of their paired adjacent normal liver tissue samples. Overall survival curves were calculated by the Kaplan-Meier method and were analyzed with the log-rank test. All analyses were performed using the SPSS version 13.0 program (SPSS). Statistical significance was defined as *P*< 0.05.

## Results

### The expression of E-cadherin, HSP70, α-SMA and p-p38 in HCC tissues

#### Real-time RT-PCR and Western blotting

**Figure 1:**
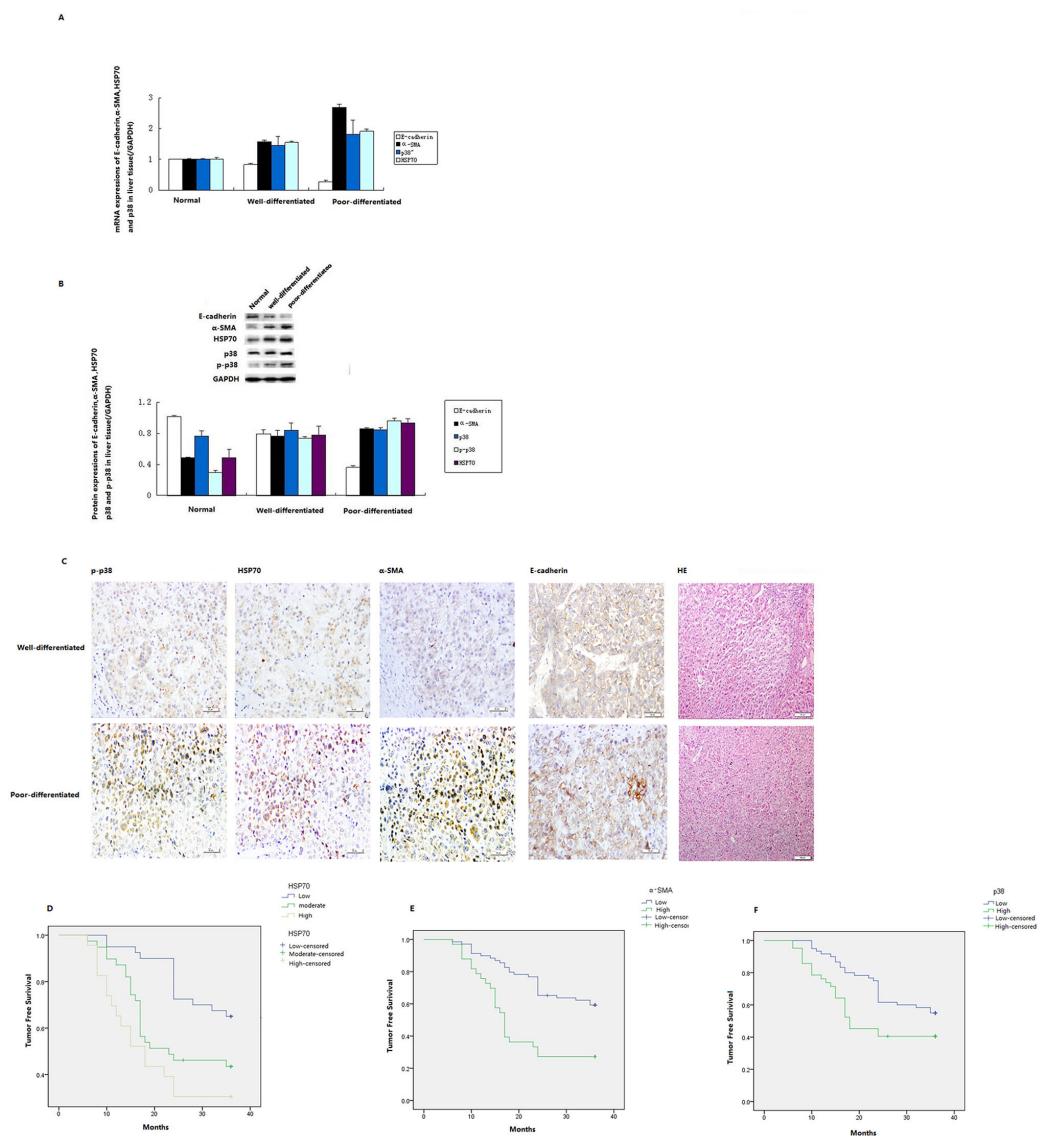
The mRNA and protein expression of E-cadherin, p-p38, HSP70 and α-SMA in tumors and the correlation between tumor-free survival and expression of HSP70, α-SMA and p-p38. (A) The mRNA expressions of E-cadherin, α-SMA and HSP70 and p38 (normalized to GADPH expression). (B) Photomicrographs of well-differentiated hepatic cancer (top panel) and poorly differentiated hepatic cancer. p-p38, HSP70 and α-SMA were detected in the cytoplasm; E-cadherin was detected in the plasmalemma. Original magnification, 200´. (C) The protein expressions of α-SMA, HSP70, p38 and p-p38 (normalized to GAPDH expression). Kaplan-Meier tumor-free survival curves for hepatic cancer patients showing that the median tumor-free survival time of patients correlated with HSP70 (D), α-SMA (E) and p-p38 (F) expression.

Frozen specimens from 60 cases of liver cancer and 17 cases of normal liver tissues were analyzed the expression of HSP70, E-cadherin, α-SMA and p38 by real-time RT-PCR and western blotting. The expressions of HSP70, E-cadherin, α-SMA and p-p38 mRNA in HCC tissue were significantly higher than in normal liver tissue (Figure 1A). Consistent with the mRNA expression, the expressions of HSP70, E-cadherin, α-SMA and p38 proteins were also elevated in HCC tissues (Figure 1B).

#### Immunohistochemistry staining results

To corroborate the gene expression data, immunohistochemical analysis was used. In the 102 paired HCC tissues, positive staining of HSP70, α-SMA and p-p38 protein was mainly found in the cytoplasm and positive staining of E-cadherin was mainly located on the plasmalemma (Figure 1C). All 102 liver cancer tissues showed positive E-cadherin staining (67.6% (69/102 low expression; 32.4% (33/102) high expression). Positive HSP70 staining was observed in 100% of HCC tissues (40 cases (39.2%) with low expression, 39 (38.2%) with moderate expression and 23 (22.5%) with high expression). Positive α-SMA protein staining in the HCC tissues was 29.4% (30/102) (91 (89.2%) with low expression and 11 (10.8%) with high expression). Positive p38 staining in the HCC tissues was 96.1% (98/102) (58 (58.8%) with high expression and 44 (41.2%) with low expression).

### The relationship between the expression of E-cadherin, HSP70, α-SMA and p-p38 in HCC and the clinicopathological characteristics and prognosis of patients

The expressions of HSP70, α-SMA, E-cadherin and p-p38/MAPK were closely related to the differentiation degree, TNM staging, lymph node metastasis and portal vein invasion of HCC (Table 1). However, the expressions of HSP70, α-SMA, E-cadherin and p-p38/MAPK were not significantly correlated with clinicopathological parameters, e.g. patient age, gender and tumor size. 

The immunohistochemistry results and follow-up information on the factors affecting liver cancer prognosis were subjected to univariate and multivariate analysis. At the end of the follow-up, the postoperative relapse-free survival time ranged from 6–36 months, and the postoperative relapse-free median survival time was 26.3 months. Kaplan-Meier survival analysis showed that the postoperative relapse-free median survival times for patients with high expression and low expression of E-cadherin protein were 30.6 and 23.1 months, respectively. The postoperative relapse-free median survival time in patients with high expression, moderate expression and low expression of HSP70 were 18.0, 23.0 and 31.3months, respectively (Log rank: P<0.05). The postoperative relapse-free median survival time in patients with high expression and low expression of α-SMA protein were 17.0 and 29.3 months, respectively (Log rank: P<0.05). The postoperative relapse-free median survival times in patients with high expression and low expression of p-p38 protein were 29.0 months and 18.0 months, respectively (Log Rank: P<0.05). As can be seen from the patient's survival function curve, the expressions of HSP70, α-SMA and p-p38 protein correlated with prognosis and survival rate (Figure 1D, 1E and 1F). In the parameters of clinical cases, the differentiation degree of liver cancer (P<0.05), TNM staging (P<0.05), lymph node metastasis (P<0.05) and portal vein invasion (P<0.05), significantly affected the average postoperative relapse-free survival time. 

The results of multivariate Cox proportional hazards model analysis of the expression of E-cadherin, HSP70, α-SMA and p-p38 protein, liver cancer differentiation, TNM staging, lymph node metastasis and portal vein invasion are shown in Table 2. The expressions of HSP70 and α-SMA protein (P<0.05) and TNM staging (P<0.05) were significantly related to prognosis. Differentiation degree (P=0.217) and lymph node metastasis (P=0.286) were not.

**Table 2:** Factors influencing survival time.

Factor	*OR*	95% CI	*P*
Degree of differentiation	1.303	0.782-2.1722	>0.05
Lymph node metastasis	0.709	0.430-1.117	>0.05
The stage of TNM	2.592	1.643-4.091	<0.05
The portal vein invasion	0.414	0.194-0.884	<0.05
Expression of E-cadherin	2.446	1.077-5.557	<0.05
Expression of α-SMA	2.401	1.278-4.513	<0.05
Expression of p-p38	0.464	0.226-0.953	<0.05
Expression of HSP70	2.486	1.118-5.528	<0.05

### Extracellular HSP70/HSP70-PCs can promote the occurrence of EMT in Huh-7 hepatoma cells

**Figure 2:**
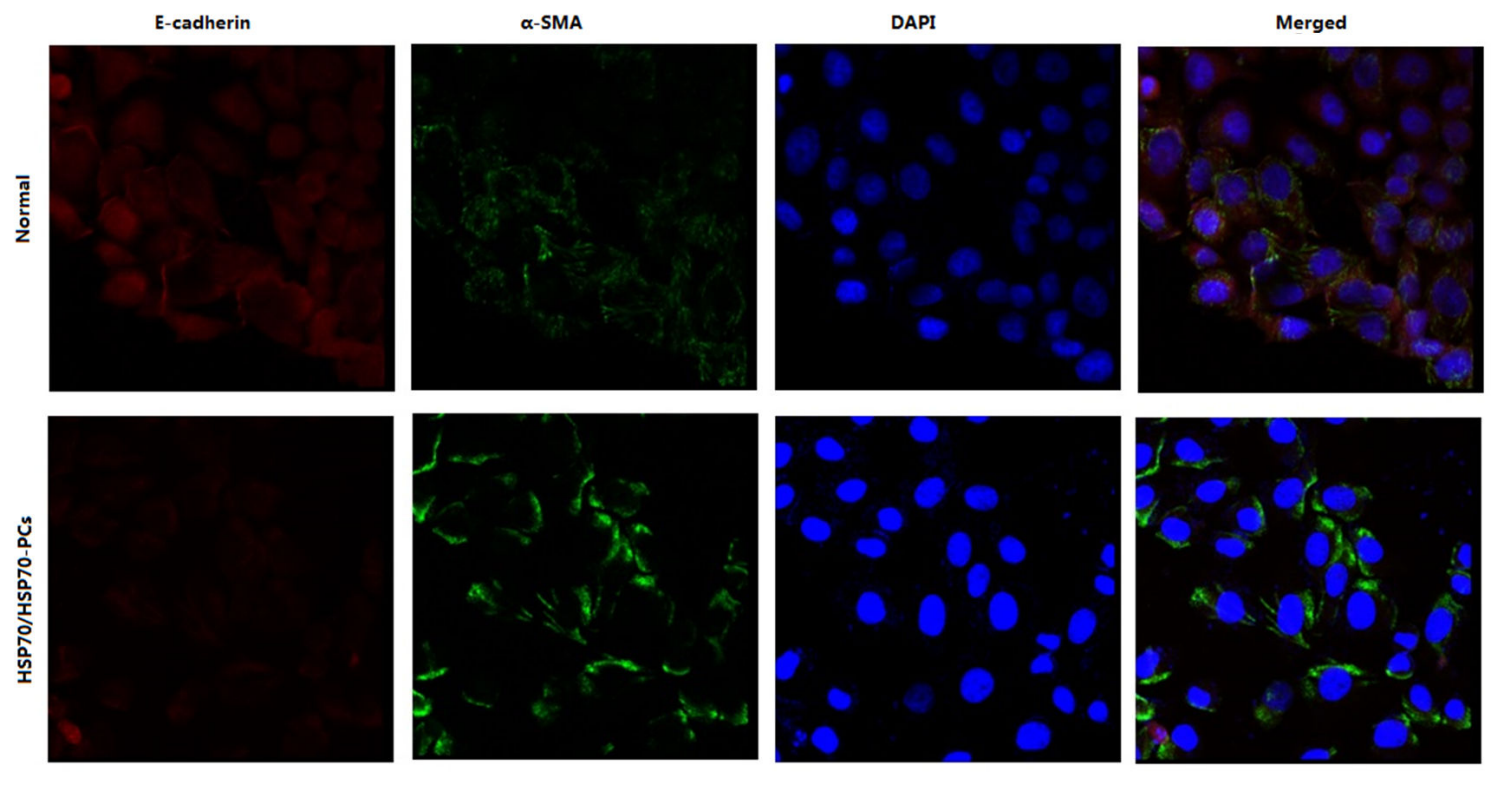
Expression of E-cadherin (red) and α-SMA (green) in Huh-7 cells observed by immunofluorescence. α-SMA was detected in the cytoplasm; E-cadherin was found in the plasmalemma and the cytoplasm. HSP70/HSP70-PCs treatment reduced the expressions of E-cadherin and promoted the expression of α-SMA.

We used the immunofluorescent (IF) staining of E-cadherin and α-SMA in Huh-7 cells. Immunostaining and confocal microscopy showed that E-cadherin was located in the plasmalemma and the cytoplasm of Huh-7 cells and α-SMA was in the cytoplasm. Treatment with HSP70/HSP70-PCs reduced the expression of E-cadherin and promoted the expression of α-SMA (Figure 2). 

**Figure 3:**
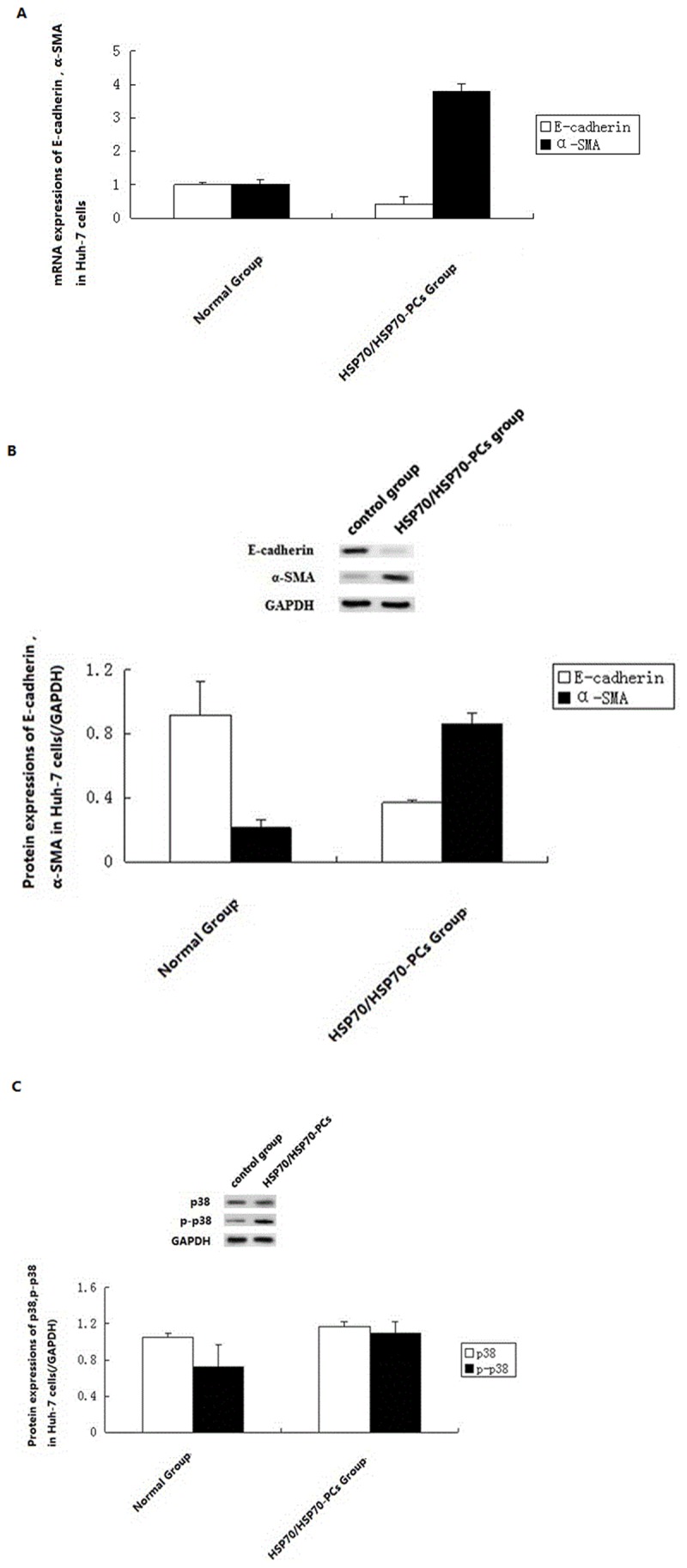
HSP70/HSP70-PCs affect the protein levels of E-cadherin , α-SMA (A), total-p38, phosphor-p38 (C) and mRNA (B) expression of E-cadherin and α-SMA. 24h after induction, cells were harvested for western blotting and real-time RT-PCR. Data are presented as means ± SD from three independent experiments (normalized to GADPH expression).

Real-time RT-PCR showed that the expression of E-cadherin mRNA in HSP70/HSP70-PCs-induced group significantly decreased, while the expression of mesothelial cell marker α-SMA mRNA significantly increased (Figure 3A). Western blotting showed that, compared with cells in the control group, after incubation with HSP70/HSP70-PCs (2μg/ml) the expression of epithelial marker E-cadherin protein in Huh-7 cells significantly decreased, while the expression of mesothelial cells marker α-SMA protein significantly increased (Figure 3B). The gradual disappearance of the epithelial cell marker and the increase in the mesenchymal cell marker indicated that HSP70/HSP70-PCs induced the cells to enter EMT.

### EMT acts through the p38/MAPK pathway

**Figure 4:**
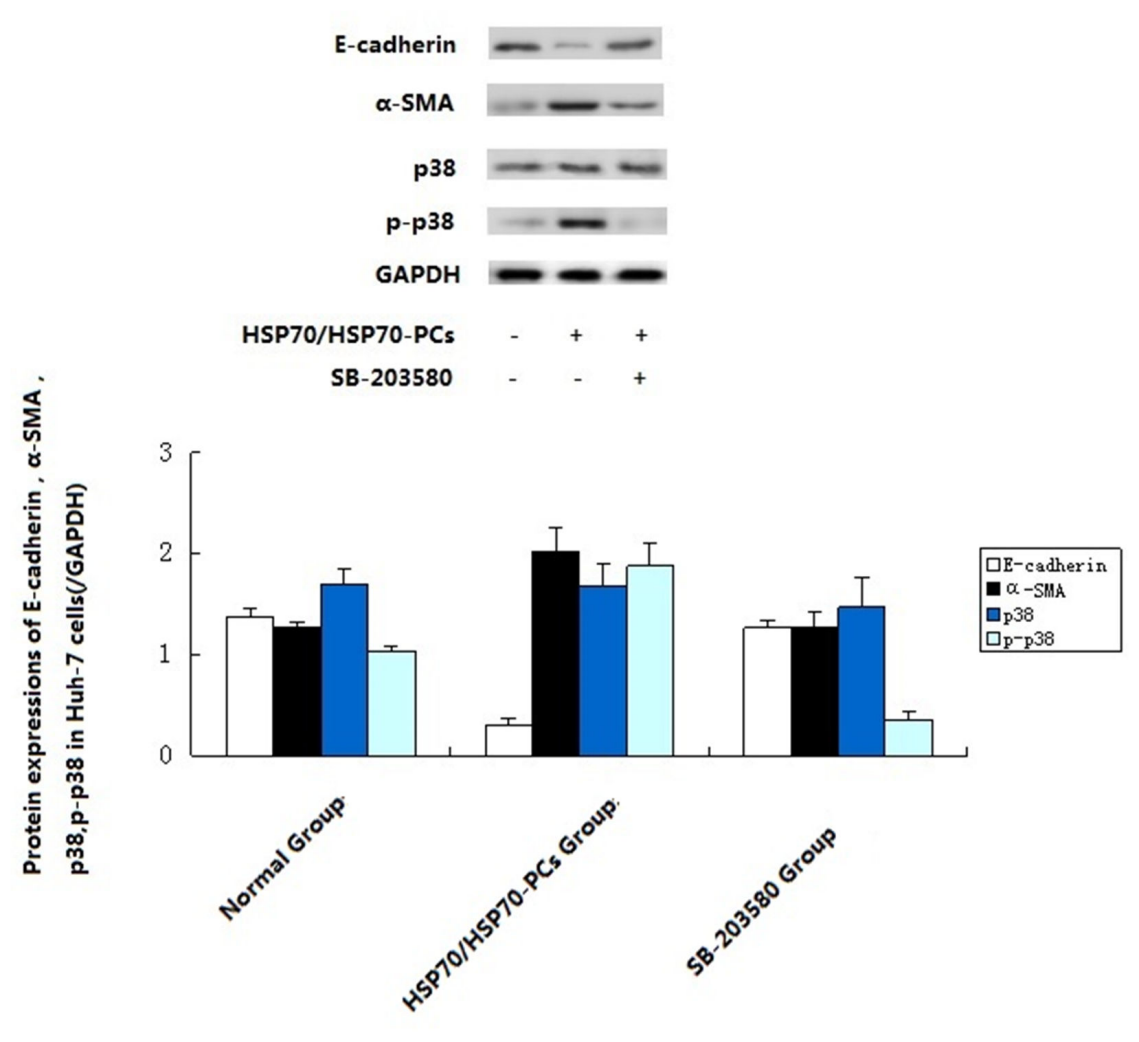
Effects of blocking the p38 signal pathway on HSP70/HSP70-PCs stimulation of E-cadherin, α-SMA , p38 and p-p38. Cells were treated with SB-203580 (10μM) for 1 h to block the p38 signal pathway and then exposed to HSP70/HSP70-PCs for 24h. Cell extracts were subjected to western blotting. Data are presented as means ± SD from three independent experiments (normalized to GADPH expression).

Compared with the control group, Huh-7 cells pre-treated with SB-203580 before exposure to eHSP70/HSP70-PCs did not undergo EMT: the expressions of E-cadherin and α-SMA did not change significantly (Figure 4). Thus, the promotion of EMT in Huh-7 cells by eHSP70/HSP70-PCs was achieved via the p38/MAPK pathway.

## Discussion

The invasion, metastasis and relapse of malignant tumor is a complex dynamic process, which is accompanied by tumor progression, including cell adhesion, cell motility, cell proliferation, extracellular matrix degradation, tumor angiogenesis, and many other aspects [17]. EMT is the molecular basis of tumor cell infiltration and metastasis [18]. After EMT in tumor cells, the decrease or absence of epithelial marker E-cadherin is an important sign. Conversely, upregulation of the mesenchymal marker α-SMA occurs during EMT [3]. Thus, the detection of these two specific cell surface markers indicates the occurrence of EMT. A variety of factors in the tumor microenvironment can promote EMT, including LPS and TGF-β [19]; thus, during the establishment and development of a tumor, the changing microenvironment is one of the most important factors for inducing EMT. HSP70 is also present in the tumor microenvironment [9] and this widespread protein family is involved in exocytosis, secretion and tumor cell necrosis in vivo [20]. In this study, western blotting showed that HSP70 was present in cell culture medium, indicating that HSP70 is released into the microenvironment. In vitro studies showed that eHSP70/HSP70-PCs play a complex and contradictory role at different stages of tumor progression [11]. In the early stage, eHSP70/HSP70-PCs cause immune suppression of tumor growth; in the advanced stage, HSP70/HSP70-PCs in the tumor microenvironment promote tumor growth through immunosuppression [11]. Walsh [12] observed that eHSP70/HSP70-PCs also directly affect the regulation and invasion of tumor cells and other biological processes. However, there was no report on the effect of eHSP70/HSP70-PCs on EMT of tumor cells. 

We explored the relationship between the expressions of HSP70, E-cadherin and α-SMA protein in HCC and clinicopathological parameters. The results suggested that HSP70 protein was expressed significantly higher in HCC than in normal liver tissue. The EMT-related protein E-cadherin was expressed at a lower level in HCC than in normal liver tissue, and the expression of EMT-related protein α-SMA in HCC was significantly higher than in normal liver tissue. The expressions of HSP70 protein, E-cadherin and α-SMA protein were associated with the occurrence of HCC. Combined with HCC grade, the expressions of HSP70 and α-SMA in poorly differentiated HCC were significantly higher than in highly differentiated HCC. α-SMA protein expression was closely related to HCC progress. The expressions of HSP70, E-cadherin and α-SMA protein were related to lymph node metastasis and TNM staging, which suggested that the three could be used as clinical stage indicators. Kaplan-Meier survival analysis and multivariate Cox proportional hazards model analysis showed that HSP70 and α-SMA protein were risk factors: the higher their expressions, the shorter the average postoperative relapse-free survival time. Thus, HSP70 and α-SMA protein may promote the proliferation of malignant cells, and their upregulated expressions result in postoperative HCC recurrence and poor prognosis. 

To further investigate the effect of eHSP70/HSP70-PCs on the occurrence of EMT in hepatoma cells and its mechanism, we treated the hepatoma cell line Huh-7 with purified HSP70/HSP70-PCs from human liver cancer tissue in vitro to observe the occurrence of EMT, with encouraging results. Activation by eHSP70/HSP70-PCs caused E-cadherin (the epithelial marker) to disappear, and α-SMA (the mesenchymal marker) to increase in Huh-7 cells. Thus, Huh-7 cells acquired a mesenchymal phenotype through EMT induced by eHSP70/HSP70-PCs. 

How do eHSP70/HSP70-PCs induce EMT in hepatoma cells? The literature indicated that the p38/MAPK signal transduction pathway might be involved in the process of EMT. The p38/MAPK signaling pathway can respond to a variety of stimuli in the tumor microenvironment, regulating changes in the biological behavior of the tumor cells, and promote proliferation and invasion [21]. Western blotting and real-time RT-PCR showed that when eHSP70/HSP70-PCs induced EMT in Huh-7 cells, the expression of p38 was upregulated significantly. Thus, p38 might be involved in the process of EMT. Using a p38 inhibitor, the changes in expressions of EMT marker proteins induced by eHSP70/HSP70-PCs could be reversed. This result showed that induction of EMT in hepatoma cells by eHSP70/HSP70-PCs might involve the p38/MAPK pathway.

## Conclusions

The expressions of HSP70, E-cadherin, α-SMA and p-p38 can be indicators of malignant potential and might be used to discriminate the malignant degree of liver cancer. Extracellular HSP70/HSP70-PCs play an important role in EMT of hepatocarcinoma via the p38/MAPK pathway. Our study provides a new theoretical and experimental basis for the pathogenesis and prevention of liver cancer invasion.
